# The Ophthalmology of the Poets

**Published:** 1896-02-15

**Authors:** 


### The Ophthalmology of the Poets.

No part of the human anatomy is more frequently and fully described by the poets than the eye, and it s amusing, if not instructive, to collect and compare some of their allusions to it.

Naturally the first thing a poet notices about the eye is its colour, and here we find many men of many minds, or as the poet Gray put it—

> Nature in various moulds has beauty cast,
> And formed the features for each different taste;
> This sighs for golden locks and azure eyes;
> That for the gloss of sable tresses dies.

But the formation of these varying tastes seems to be influenced by environment, and poets have a happy faculty—due no doubt to their impressionable natures —for seeing beauty in the colour of the eye which prevails in their neighbourhood.

Tennyson, for instance, living among the fair-haired and blue-eyed Saxons of the South of England, describes many of his heroes and heroines of this type· Margaret, one of his earliest heroines, has blue eyes; Katie Willows, in "The Brook," has "eyes a bashful azure." "A prince I was, blue-eyed and fair in face," says the adventurous hero in "The Princess." Maud's lover casually mentions to her, "violets blue as your eyes," and Enid, in "Idylls of the King," has "meek blue eyes." So also Tom Ingoldsby, the home of whose legends is almost always in Kent, speaks of "the sky, which had been of a dye as bright and as blue as your lady-love's eye."

Robert Browning, on the other hand, lived chiefly in Italy, and in his somewhat rare descriptions of physical characteristics certainly speaks of black eyes oftener than any other, as in "The Lost Mistress" we have "each glance of the eye so bright and black," and the poor bride, in "The Statue and the Bust," has "eyes of the blackest black."

Tom Moore did not seem particular as to the exact colour so long as the eyes were dark; he had never a word of praise for any other.

Lord Byron, as might have been expected of such a roving character, was an admirer of all colours:—

> An eye's an eye, and whether black or blue
> Is no great matter, so 'tis in request;
> 'Tis nonsense to dispute about a hue,
> The kindest may be taken as a test.
> The fair sex should be always fair, and no man
> Till thirty should perceive there's a plain woman.

Still his preference seems to have been for dark eyes, of which he certainly saw most in Italy and Greece:—

> Her eye (I'm very fond of handsome eyes)
> Was large and dark, suppressing half its fire
> Until she spoke.

And again:—

> She walks in beauty, like the night
> Of cloudless climes and starry skies;
> And all that's best of dark and bright
> Meet in her aspect and her eyes.

It is exceedingly difficult to say what Shakespeare's preference was. On the whole, I think it was, like Romeo's, for "a white wench's black eye," but he refers to every possible colour except, I believe, brown; and this calls to mind the curious fact that brown eyes are seldom or never mentioned by poets. In fact, I only know one reference to them—"Maiden with the meek brown eyes"—which occurs in one of Longfellow's shorter poems.

Grey eyes are often mentioned, especially by Shakespeare and Tennyson. Julia, in "Two Gentlemen of Verona," has "eyes as grey as glass." Olivia, in "Twelfth Night," in giving the divers schedules of her beauty, includes "item, two grey eyes."

Tennyson's Miller has twinkling grey eyes, and Enoch Arden has "large grey eyes and weather-beaten face," which calls to mind Wordsworth's "noticeable man with large grey eyes." None of Tennyson's womenkind have grey eyes, so far as I know.

The colour of the eyes is constantly referred to by poets in connection with the character they are supposed to indicate. Grey eyes, for instance, are often taken as connected with good nature and a sense of humour. Tennyson's Miller, just referred to, is an example:—

> In yonder chair I see him sit,
> Three fingers round the old silver cup;
> I see his grey eyes twinkle yet
> At his own jest. Grey eyes lit up
> With summer lightnings of a soul
> So full of summer warmth, so glad,
> So healthy, sound, and clear, and whole,
> His memory scarce can make me sad.

Black eyes are associated with mischief and witchcraft. In one of the Ingoldsby legends the hero, seeing a witch, succumbs to "a single glance of that coal-black eye." Tennyson describes "airy fairy Lilian" as "glancing with black beaded eyes." According to Moore, Mohammedans attribute black eyes to the Houris in Paradise:—

> A Persian's heaven is easily made,
> 'Tis but black eyes and lemonade.

Green eyes have but few admirers, and are not associated with very desirable traits of character, as in Burns:—

> Sic a wife as Willie had, I wadna' gie a button for her;
> She has an e'e—she has but ane; the cat has twa the very colour.

Red or fiery eyes are several times mentioned by Shakespeare as indicating great anger or malice, as, when Mercutio is slain, Romeo cries,—

> Away to heaven respective lenity,
> And fire-eyed fury be my conduct now.

Again, in "Henry VI.":—

> His sparkling eyes, replete with wrathful fire,
> More dazzled and drove back his enemies
> Than midday sun.

One of King John's nobles is seen "with eyes as red as new enkindled fire." In "Henry VI." we have:—

> Beaufort's red sparkling eyes blab his heart's malice.

And Cominius, speaking of Coriolanus, says:—

> I tell you, he does sit in gold; his eye
> Red as 'twould burn Rome.

